# 1-(3-Meth­oxy­phen­yl)-2-(phenyl­sulfon­yl)ethan-1-one

**DOI:** 10.1107/S1600536812032795

**Published:** 2012-07-28

**Authors:** Sammer Yousuf, Sarosh Iqbal, Nida Ambreen, Khalid M. Khan

**Affiliations:** a H. E. J. Research Institute of Chemistry, International Center for Chemical and Biological Sciences, University of Karachi, Karachi 75270, Pakistan

## Abstract

In the title compound, C_15_H_14_O_4_S, the dihedral angle between the benzene and phenyl rings is 88.74 (10)°. In the crystal, mol­ecules are linked into a three-dimensional network by C—H⋯O hydrogen bonds and π–π stacking inter­actions [centroid–centroid distances = 3.6092 (13)–3.8651 (13) Å].

## Related literature

For the biological activity of sulfone compounds, see: Dawood *et al.* (2010 ▶); Suryakiran *et al.* (2007 ▶); Siddiq *et al.* (2005 ▶); Lai *et al.* (2005 ▶). For related structures, see: Yousuf *et al.* (2012 ▶); Billing *et al.* (2006 ▶); Pei *et al.* (2005 ▶); Gu *et al.* (2004 ▶).
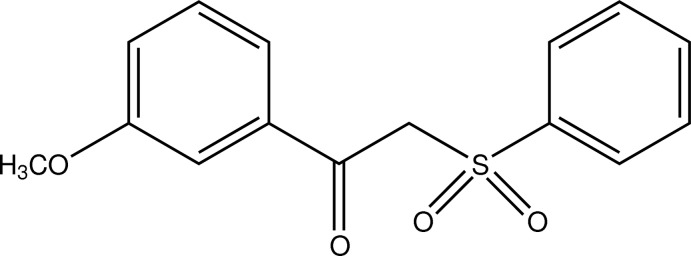


## Experimental

### 

#### Crystal data

C_15_H_14_O_4_S
*M* *_r_* = 290.32
Triclinic, 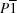

*a* = 7.1290 (6) Å
*b* = 9.6101 (8) Å
*c* = 10.6999 (9) Å
α = 101.787 (2)°
β = 102.550 (2)°
γ = 95.879 (2)°
*V* = 692.13 (10) Å^3^
*Z* = 2
Mo *K*α radiation
μ = 0.24 mm^−1^
*T* = 298 K
0.30 × 0.12 × 0.07 mm

#### Data collection

Bruker SMART APEX CCD area-detector diffractometer
Absorption correction: multi-scan (*SADABS*; Bruker, 2000 ▶) *T*_min_ = 0.931, *T*_max_ = 0.983
7866 measured reflections
2577 independent reflections
2135 reflections with *I* > 2σ(*I*)
*R*_int_ = 0.026

#### Refinement

*R*[*F* ^2^ > 2σ(*F* ^2^)] = 0.043
*wR*(*F* ^2^) = 0.108
*S* = 1.06
2577 reflections
181 parameters
H-atom parameters constrained
Δρ_max_ = 0.24 e Å^−3^
Δρ_min_ = −0.29 e Å^−3^

### 

Data collection: *SMART* (Bruker, 2000 ▶); cell refinement: *SAINT* (Bruker, 2000 ▶); data reduction: *SAINT*; program(s) used to solve structure: *SHELXS97* (Sheldrick, 2008 ▶); program(s) used to refine structure: *SHELXL97* (Sheldrick, 2008 ▶); molecular graphics: *SHELXTL* (Sheldrick, 2008 ▶); software used to prepare material for publication: *SHELXTL*, *PARST* (Nardelli, 1995 ▶) and *PLATON* (Spek, 2009 ▶).

## Supplementary Material

Crystal structure: contains datablock(s) global, I. DOI: 10.1107/S1600536812032795/rz2794sup1.cif

Structure factors: contains datablock(s) I. DOI: 10.1107/S1600536812032795/rz2794Isup2.hkl

Supplementary material file. DOI: 10.1107/S1600536812032795/rz2794Isup3.cml

Additional supplementary materials:  crystallographic information; 3D view; checkCIF report

## Figures and Tables

**Table 1:** Hydrogen-bond geometry (Å, °)

*D*—H⋯*A*	*D*—H	H⋯*A*	*D*⋯*A*	*D*—H⋯*A*
C7—H7*A*⋯O2^i^	0.97	2.54	3.411 (3)	149
C7—H7*B*⋯O1^ii^	0.97	2.37	3.334 (3)	171
C11—H11*A*⋯O2^iii^	0.93	2.48	3.317 (3)	150
C15—H15*A*⋯O3^iv^	0.96	2.47	3.413 (3)	167

Symmetry codes: (i) 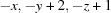; (ii) 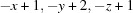; (iii) 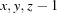; (iv) 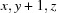.

**Fig. 1.**
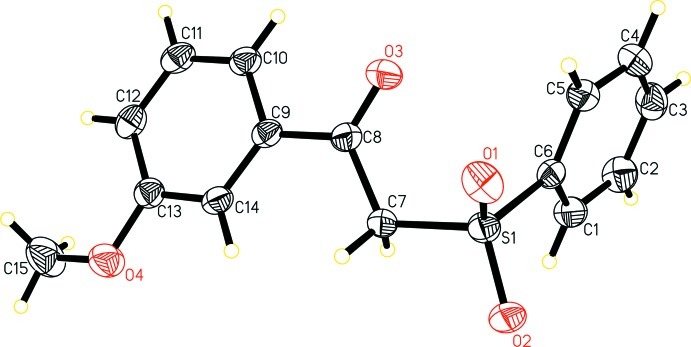
The molecular structure of the title compound with displacement ellipsoids drawn at the 30% probability level.

**Fig. 2.**
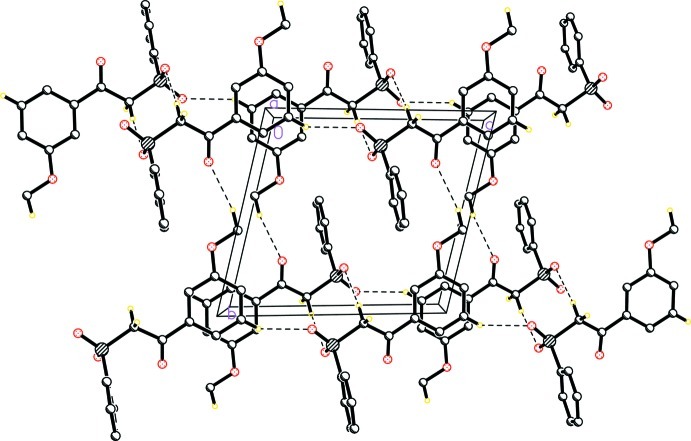
The crystal packing of the title compound. Only hydrogen atoms involved in hydrogen bonding (dashed lines) are shown.

## supplementary crystallographic information

### Comment

Sulfone derivatives represent an important class of organic compounds and are known to have a wide range of biological activities including antiviral, antitubercular, anti-human rennin and antimicrobial activities (Dawood *et al.*, 2010; Suryakiran *et al.*, 2007; Siddiq *et al.*, 2005; Lai *et al.*, 2005). The title compound was obtained as part of our ongoing research to synthesize novel sulfone derivatives in order to study their different biological activities and structure-activity relationships.

In the title compound, C_15_H_14_O_4_S, the phenyl (C1–C6) and benzene (C9–C14) rings form a dihedral angle of 88.74 (10)°. The bond dimensions and angles are similar to those found in structurally related compounds (Yousuf *et al.*, 2012; Billing *et al.*, 2006; Pei *et al.*, 2005; Gu *et al.*, 2004). In the crystal structure (Fig. 2), the molecules are linked to form a three-dimensional network by C7–H7A···O2, C7–H7B···O1, C11–H11A···O2 and C15–H15A···O3 hydrogen interactions (Table 1) and by π···π stacking interactions [Cg1···Cg1^i^, 3.8651 (13) Å; Cg2···Cg2^ii^, 3.6092 (12) Å; Cg2···Cg2^iii^, 3.7240 (13) Å. Cg1 and Cg2 are the centroids of the C1–C6 and C9–C14 rings, respectively. Symmetry codes: (i) -x, 1-y, 1-z; (ii) -x, 2-y, -z; (iii) 1-x, 2-y, -z].

### Experimental

In a 50 ml round-bottomed flask, benzene sulfonyl chloriode (6 mmol), water (15 ml), sodium bicarbonate (0.840 g, 10 mmol) and sodium sulfite (1.26 g, 10 mmol) were added and refluxed for about 4–7 h. The progress of the reaction was monitored by TLC until complete disappearance of the starting material indicated the formation of sodium sulfinate salt. 2-Bromo-3'-methoxyacetophenone (2 mmol) in ethanol (7 ml) was then added and the mixture refluxed for 7 h till completion of reaction (TLC analysis). After cooling, the reaction mixture was neutralized by adding dilute HCl. The precipitate obtained was filtered and recrystallized from ethanol to obtain crystals of 1-(2-methoxyphenyl)-3-(phenylsulfonyl)-1-ethanone (0.49 g, 84% yield) suitable for single-crystal X-ray diffraction studies.

### Refinement

H atoms on methyl, methylene and methine carbon atoms were positioned geometrically with C—H = 0.96 Å, 0.97 Å, and 0.93 Å, respectively, and constrained to ride on their parent atoms with *U*_iso_(H)= 1.2*U*_eq_(C) or 1.5*U*_eq_(C) for methyl H atoms.

### Crystal data

**Table:**

C_15_H_14_O_4_S	*Z* = 2
*M**_r_* = 290.32	*F*(000) = 304
Triclinic, *P*1	*D*_x_ = 1.393 Mg m^−^^3^
Hall symbol: -P 1	Mo *K*α radiation, λ = 0.71073 Å
*a* = 7.1290 (6) Å	Cell parameters from 2142 reflections
*b* = 9.6101 (8) Å	θ = 2.2–24.2°
*c* = 10.6999 (9) Å	µ = 0.24 mm^−^^1^
α = 101.787 (2)°	*T* = 298 K
β = 102.550 (2)°	Plate, colourless
γ = 95.879 (2)°	0.30 × 0.12 × 0.07 mm
*V* = 692.13 (10) Å^3^	

### Data collection

**Table:**

Bruker SMART APEX CCD area-detector diffractometer	2577 independent reflections
Radiation source: fine-focus sealed tube	2135 reflections with *I* > 2σ(*I*)
Graphite monochromator	*R*_int_ = 0.026
ω scan	θ_max_ = 25.5°, θ_min_ = 2.0°
Absorption correction: multi-scan (*SADABS*; Bruker, 2000)	*h* = −8→8
*T*_min_ = 0.931, *T*_max_ = 0.983	*k* = −11→11
7866 measured reflections	*l* = −12→12

### Refinement

**Table:**

Refinement on *F*^2^	Primary atom site location: structure-invariant direct methods
Least-squares matrix: full	Secondary atom site location: difference Fourier map
*R*[*F*^2^ > 2σ(*F*^2^)] = 0.043	Hydrogen site location: inferred from neighbouring sites
*wR*(*F*^2^) = 0.108	H-atom parameters constrained
*S* = 1.06	*w* = 1/[σ^2^(*F*_o_^2^) + (0.0521*P*)^2^ + 0.113*P*] where *P* = (*F*_o_^2^ + 2*F*_c_^2^)/3
2577 reflections	(Δ/σ)_max_ < 0.001
181 parameters	Δρ_max_ = 0.24 e Å^−^^3^
0 restraints	Δρ_min_ = −0.29 e Å^−^^3^

### Special details

**Table:**

Geometry. All e.s.d.'s (except the e.s.d. in the dihedral angle between two l.s. planes) are estimated using the full covariance matrix. The cell e.s.d.'s are taken into account individually in the estimation of e.s.d.'s in distances, angles and torsion angles; correlations between e.s.d.'s in cell parameters are only used when they are defined by crystal symmetry. An approximate (isotropic) treatment of cell e.s.d.'s is used for estimating e.s.d.'s involving l.s. planes.
Refinement. Refinement of *F*^2^ against ALL reflections. The weighted *R*-factor *wR* and goodness of fit *S* are based on *F*^2^, conventional *R*-factors *R* are based on *F*, with *F* set to zero for negative *F*^2^. The threshold expression of *F*^2^ > σ(*F*^2^) is used only for calculating *R*-factors(gt) *etc*. and is not relevant to the choice of reflections for refinement. *R*-factors based on *F*^2^ are statistically about twice as large as those based on *F*, and *R*- factors based on ALL data will be even larger.

### Fractional atomic coordinates and isotropic or equivalent isotropic displacement parameters (Å^2^)

**Table:**

	*x*	*y*	*z*	*U*_iso_*/*U*_eq_	
S1	0.22994 (8)	0.83326 (5)	0.47049 (5)	0.04481 (19)	
O1	0.4151 (2)	0.78524 (16)	0.49651 (15)	0.0599 (4)	
O2	0.1737 (2)	0.91873 (16)	0.57906 (14)	0.0597 (4)	
O3	0.2985 (3)	0.75030 (17)	0.20387 (17)	0.0726 (5)	
O4	0.2706 (3)	1.33137 (17)	0.10446 (16)	0.0669 (5)	
C1	−0.1457 (3)	0.7024 (2)	0.3856 (2)	0.0519 (5)	
H1B	−0.1776	0.7931	0.4136	0.062*	
C2	−0.2891 (3)	0.5848 (3)	0.3324 (2)	0.0608 (6)	
H2B	−0.4189	0.5960	0.3243	0.073*	
C3	−0.2412 (4)	0.4505 (3)	0.2909 (2)	0.0600 (6)	
H3A	−0.3387	0.3714	0.2556	0.072*	
C4	−0.0507 (4)	0.4333 (2)	0.3017 (2)	0.0579 (6)	
H4A	−0.0194	0.3424	0.2736	0.069*	
C5	0.0956 (3)	0.5502 (2)	0.3541 (2)	0.0489 (5)	
H5A	0.2251	0.5388	0.3605	0.059*	
C6	0.0467 (3)	0.6838 (2)	0.39682 (18)	0.0411 (5)	
C7	0.2252 (3)	0.9421 (2)	0.35511 (19)	0.0432 (5)	
H7A	0.0977	0.9713	0.3363	0.052*	
H7B	0.3193	1.0286	0.3958	0.052*	
C8	0.2679 (3)	0.8732 (2)	0.2256 (2)	0.0462 (5)	
C9	0.2659 (3)	0.9613 (2)	0.12651 (19)	0.0411 (5)	
C10	0.2567 (3)	0.8914 (2)	−0.0027 (2)	0.0469 (5)	
H10A	0.2537	0.7923	−0.0250	0.056*	
C11	0.2523 (3)	0.9691 (3)	−0.0966 (2)	0.0520 (6)	
H11A	0.2465	0.9222	−0.1828	0.062*	
C12	0.2562 (3)	1.1161 (3)	−0.0658 (2)	0.0506 (5)	
H12A	0.2527	1.1676	−0.1308	0.061*	
C13	0.2653 (3)	1.1867 (2)	0.0624 (2)	0.0462 (5)	
C14	0.2706 (3)	1.1092 (2)	0.1588 (2)	0.0444 (5)	
H14A	0.2773	1.1564	0.2450	0.053*	
C15	0.2437 (5)	1.4133 (3)	0.0078 (3)	0.0859 (9)	
H15A	0.2509	1.5127	0.0498	0.129*	
H15B	0.1186	1.3794	−0.0523	0.129*	
H15C	0.3434	1.4033	−0.0395	0.129*	

### Atomic displacement parameters (Å^2^)

**Table:**

	*U*^11^	*U*^22^	*U*^33^	*U*^12^	*U*^13^	*U*^23^
S1	0.0551 (3)	0.0411 (3)	0.0331 (3)	0.0025 (2)	0.0064 (2)	0.0042 (2)
O1	0.0535 (9)	0.0548 (10)	0.0617 (10)	0.0037 (7)	−0.0026 (7)	0.0119 (8)
O2	0.0897 (12)	0.0524 (9)	0.0327 (8)	0.0070 (8)	0.0155 (8)	0.0016 (7)
O3	0.1229 (16)	0.0439 (10)	0.0609 (11)	0.0186 (10)	0.0449 (10)	0.0079 (8)
O4	0.0959 (13)	0.0517 (10)	0.0565 (10)	0.0158 (9)	0.0211 (9)	0.0156 (8)
C1	0.0570 (14)	0.0500 (13)	0.0490 (13)	0.0111 (11)	0.0152 (10)	0.0088 (10)
C2	0.0487 (13)	0.0663 (16)	0.0661 (16)	0.0063 (12)	0.0135 (11)	0.0141 (13)
C3	0.0601 (15)	0.0551 (14)	0.0585 (15)	−0.0078 (11)	0.0097 (12)	0.0125 (11)
C4	0.0695 (16)	0.0400 (12)	0.0620 (15)	0.0043 (11)	0.0178 (12)	0.0071 (11)
C5	0.0520 (12)	0.0441 (12)	0.0503 (13)	0.0053 (10)	0.0160 (10)	0.0077 (10)
C6	0.0506 (12)	0.0410 (11)	0.0317 (10)	0.0041 (9)	0.0116 (9)	0.0084 (8)
C7	0.0530 (12)	0.0370 (11)	0.0372 (11)	0.0017 (9)	0.0128 (9)	0.0041 (9)
C8	0.0522 (12)	0.0428 (12)	0.0421 (12)	0.0022 (10)	0.0175 (10)	0.0029 (9)
C9	0.0354 (10)	0.0471 (12)	0.0380 (11)	0.0028 (9)	0.0109 (8)	0.0035 (9)
C10	0.0464 (12)	0.0487 (12)	0.0402 (11)	0.0018 (10)	0.0117 (9)	0.0000 (9)
C11	0.0475 (12)	0.0672 (16)	0.0345 (11)	0.0034 (11)	0.0083 (9)	0.0011 (10)
C12	0.0452 (12)	0.0674 (15)	0.0403 (12)	0.0072 (11)	0.0094 (9)	0.0167 (11)
C13	0.0437 (11)	0.0486 (13)	0.0464 (12)	0.0070 (9)	0.0121 (9)	0.0102 (10)
C14	0.0462 (11)	0.0493 (12)	0.0371 (11)	0.0070 (9)	0.0143 (9)	0.0045 (9)
C15	0.127 (3)	0.0563 (17)	0.0755 (19)	0.0147 (17)	0.0154 (17)	0.0275 (14)

### Geometric parameters (Å, º)

**Table:**

S1—O1	1.4317 (16)	C7—C8	1.517 (3)
S1—O2	1.4371 (15)	C7—H7A	0.9700
S1—C6	1.760 (2)	C7—H7B	0.9700
S1—C7	1.771 (2)	C8—C9	1.485 (3)
O3—C8	1.208 (2)	C9—C14	1.388 (3)
O4—C13	1.365 (3)	C9—C10	1.393 (3)
O4—C15	1.415 (3)	C10—C11	1.366 (3)
C1—C2	1.376 (3)	C10—H10A	0.9300
C1—C6	1.384 (3)	C11—C12	1.379 (3)
C1—H1B	0.9300	C11—H11A	0.9300
C2—C3	1.379 (3)	C12—C13	1.385 (3)
C2—H2B	0.9300	C12—H12A	0.9300
C3—C4	1.368 (3)	C13—C14	1.386 (3)
C3—H3A	0.9300	C14—H14A	0.9300
C4—C5	1.383 (3)	C15—H15A	0.9600
C4—H4A	0.9300	C15—H15B	0.9600
C5—C6	1.379 (3)	C15—H15C	0.9600
C5—H5A	0.9300		
			
O1—S1—O2	117.84 (10)	S1—C7—H7B	108.3
O1—S1—C6	109.38 (10)	H7A—C7—H7B	107.4
O2—S1—C6	108.11 (10)	O3—C8—C9	121.74 (19)
O1—S1—C7	109.20 (10)	O3—C8—C7	120.8 (2)
O2—S1—C7	104.93 (9)	C9—C8—C7	117.46 (18)
C6—S1—C7	106.79 (9)	C14—C9—C10	119.73 (19)
C13—O4—C15	117.60 (19)	C14—C9—C8	122.06 (18)
C2—C1—C6	119.1 (2)	C10—C9—C8	118.21 (18)
C2—C1—H1B	120.5	C11—C10—C9	119.7 (2)
C6—C1—H1B	120.5	C11—C10—H10A	120.2
C1—C2—C3	120.3 (2)	C9—C10—H10A	120.2
C1—C2—H2B	119.9	C10—C11—C12	121.1 (2)
C3—C2—H2B	119.9	C10—C11—H11A	119.5
C4—C3—C2	120.2 (2)	C12—C11—H11A	119.5
C4—C3—H3A	119.9	C11—C12—C13	119.7 (2)
C2—C3—H3A	119.9	C11—C12—H12A	120.2
C3—C4—C5	120.4 (2)	C13—C12—H12A	120.2
C3—C4—H4A	119.8	O4—C13—C12	124.9 (2)
C5—C4—H4A	119.8	O4—C13—C14	115.31 (19)
C6—C5—C4	119.0 (2)	C12—C13—C14	119.8 (2)
C6—C5—H5A	120.5	C13—C14—C9	119.98 (19)
C4—C5—H5A	120.5	C13—C14—H14A	120.0
C5—C6—C1	121.0 (2)	C9—C14—H14A	120.0
C5—C6—S1	120.11 (16)	O4—C15—H15A	109.5
C1—C6—S1	118.89 (16)	O4—C15—H15B	109.5
C8—C7—S1	115.89 (15)	H15A—C15—H15B	109.5
C8—C7—H7A	108.3	O4—C15—H15C	109.5
S1—C7—H7A	108.3	H15A—C15—H15C	109.5
C8—C7—H7B	108.3	H15B—C15—H15C	109.5
			
C6—C1—C2—C3	0.0 (3)	S1—C7—C8—C9	−179.81 (14)
C1—C2—C3—C4	−0.4 (4)	O3—C8—C9—C14	−166.6 (2)
C2—C3—C4—C5	0.0 (4)	C7—C8—C9—C14	14.8 (3)
C3—C4—C5—C6	0.8 (3)	O3—C8—C9—C10	14.1 (3)
C4—C5—C6—C1	−1.2 (3)	C7—C8—C9—C10	−164.46 (18)
C4—C5—C6—S1	177.26 (16)	C14—C9—C10—C11	−0.1 (3)
C2—C1—C6—C5	0.8 (3)	C8—C9—C10—C11	179.21 (18)
C2—C1—C6—S1	−177.68 (17)	C9—C10—C11—C12	−0.2 (3)
O1—S1—C6—C5	−10.3 (2)	C10—C11—C12—C13	0.2 (3)
O2—S1—C6—C5	−139.83 (17)	C15—O4—C13—C12	7.2 (3)
C7—S1—C6—C5	107.72 (18)	C15—O4—C13—C14	−173.1 (2)
O1—S1—C6—C1	168.16 (16)	C11—C12—C13—O4	179.8 (2)
O2—S1—C6—C1	38.66 (18)	C11—C12—C13—C14	0.0 (3)
C7—S1—C6—C1	−73.79 (18)	O4—C13—C14—C9	179.97 (18)
O1—S1—C7—C8	56.31 (17)	C12—C13—C14—C9	−0.3 (3)
O2—S1—C7—C8	−176.48 (15)	C10—C9—C14—C13	0.3 (3)
C6—S1—C7—C8	−61.87 (17)	C8—C9—C14—C13	−178.97 (18)
S1—C7—C8—O3	1.6 (3)		

### Hydrogen-bond geometry (Å, º)

**Table:**

*D*—H···*A*	*D*—H	H···*A*	*D*···*A*	*D*—H···*A*
C7—H7*A*···O2^i^	0.97	2.54	3.411 (3)	149
C7—H7*B*···O1^ii^	0.97	2.37	3.334 (3)	171
C11—H11*A*···O2^iii^	0.93	2.48	3.317 (3)	150
C15—H15*A*···O3^iv^	0.96	2.47	3.413 (3)	167

Symmetry codes: (i) −*x*, −*y*+2, −*z*+1; (ii) −*x*+1, −*y*+2, −*z*+1; (iii) *x*, *y*, *z*−1; (iv) *x*, *y*+1, *z*.
